# Safety profile and potential clinical risks of xanomeline and trospium chloride: A real-world pharmacovigilance study using FAERS^☆^

**DOI:** 10.1016/j.neurot.2026.e00930

**Published:** 2026-05-27

**Authors:** Zhuocheng Bao, Yakun Liu, Yuxiang Liu

**Affiliations:** a The Fifth Clinical Medical College of Shanxi Medical University, Taiyuan, China; b Department of Psychiatry, The First Hospital of Shanxi Medical University, Taiyuan, Shanxi, China; c Shanxi Key Laboratory of Artificial Intelligence Assisted Diagnosis and Treatment for Mental Disorder, Taiyuan, Shanxi, China; d Department of Nephrology, Shanxi Provincial People's Hospital (The Fifth Clinical Medical College of Shanxi Medical University), Taiyuan, Shanxi, China

**Keywords:** Schizophrenia, Xanomeline and trospium chloride, FAERS, Adverse events, Phase-IV study

## Abstract

Xanomeline and trospium chloride, the first FDA-approved oral cholinergic therapy for adults with schizophrenia in decades, represents a mechanistic shift in antipsychotic treatment, yet its real-world safety profile remains incompletely defined. Using the FDA Adverse Event Reporting System (FAERS), we conducted a retrospective pharmacovigilance study to characterize early postmarketing adverse events (AEs), prioritize clinically relevant signals, and assess subgroup heterogeneity and time-to-onset (TTO) patterns. We identified 1416 reports in which this combination was the primary suspected drug, comprising 2785 AE records. Disproportionality analyses were performed to assess drug-AE associations. The most prominent signal domain involved gastrointestinal disorders (n = 999, ROR 6.03, PRR 4.22, IC 2.08, EBGM 4.22). Beyond expected labeled events, unexpected signals such as tremor, suicidal ideation, and sedation were also observed. Clinical prioritization indicated that all signals were of low to moderate priority. Subgroup analyses largely preserved the overall safety profile, although some heterogeneity was observed across strata. Most AEs occurred within the first month of treatment (83.15%), with a median onset of 8 days, and onset was earlier in women than in men (6 vs 9 days, log-rank p = 0.0057). Weibull analyses consistently indicated an early-failure pattern, suggesting that risk decreased over time. Overall, the early postmarketing safety profile of xanomeline and trospium chloride was dominated by gastrointestinal intolerance and peripheral anticholinergic events, with additional neuropsychiatric, autonomic, and motor signals warranting continued monitoring, particularly early in treatment and in women and patients at increased risk of gastrointestinal or urinary complications.

## Graphical abstract

**Figure:**
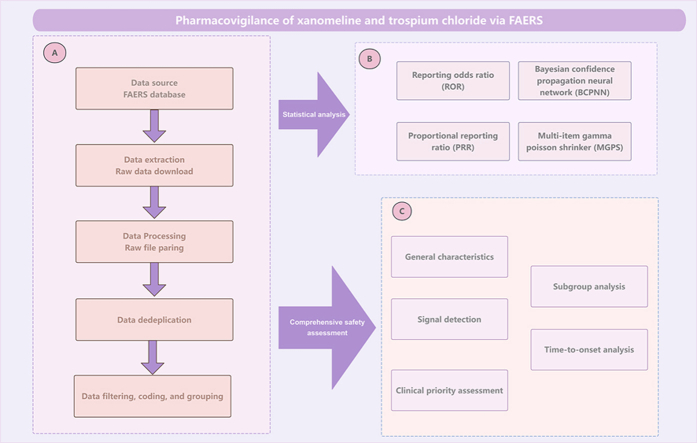

## Introduction

Schizophrenia is a severe and highly heterogeneous psychiatric disorder that typically emerges in late adolescence or early adulthood and affects approximately 24 million people worldwide [1,2]. Its course is chronic and progressive, often accompanied by relapse, hospitalization, and a high risk of death [3]. As a major contributor to the global burden of disease, schizophrenia imposes a substantial public health burden, with life expectancy reduced by 15–30 years [[4], [5], [6]]. In addition, the lifetime risk of suicide may be as high as 4.9% [7].

Since the 1950s, the pharmacological treatment of schizophrenia has been largely guided by the dopamine hypothesis. Most currently available antipsychotics act as dopamine D2 receptor antagonists or partial agonists. Although these agents are effective in reducing positive symptoms, their therapeutic limitations are well recognized. Approximately four out of five people continue to experience residual positive symptoms despite adequate dosing and treatment duration [8], and no approved antipsychotic has demonstrated consistent efficacy for negative or cognitive symptoms, which are major determinants of long term functional impairment and disability [[9], [10], [11]]. Antipsychotic therapy is also associated with a broad spectrum of adverse events (AEs), including motor symptoms (drug-induced parkinsonism [DIP], akathisia, tardive dyskinesia [TD]), hyperprolactinemia, sedation, and metabolic disturbances [12]. These AEs are key factors driving high non-adherence rates of 40%–50% and subsequent relapses [13]. Therefore, developing new therapies with novel mechanisms to address this unmet medical need is particularly urgent.

Recent research has shifted toward non-dopaminergic targets, with growing interest in muscarinic modulation of central circuits [14]. Acetylcholine is a key neurotransmitter that broadly modulates the central nervous system via muscarinic receptors, an action that incorporates dopaminergic, GABAergic, and glutamatergic signaling [[15], [16], [17]]. These biological properties make the cholinergic system a high potential target for the treatment of various neurological and psychiatric disorders.

Xanomeline, a synthetic derivative of arecoline, is a dual M1 and M4 preferring muscarinic receptor agonist without direct or indirect antagonism at dopamine D2 receptors [18,19]. Preclinical studies indicate that xanomeline preferentially inhibits dopaminergic neurons in the mesolimbic pathway relative to those projecting to the striatum, suggesting a potentially more rapid onset of antipsychotic effect while minimizing extrapyramidal liability [20]. However, xanomeline used as monotherapy causes peripheral cholinergic reactions. To address this limitation, the combination formulation KarXT (xanomeline and trospium chloride) was developed. Trospium chloride is an oral muscarinic receptor antagonist that struggles to cross the blood-brain barrier. It acts predominantly in peripheral tissues to counteract xanomeline related cholinergic AEs, a strategy intended to improve overall tolerability [21].

In pivotal EMERGENT trials, KarXT improved positive and negative symptoms with a generally manageable safety profile [[22], [23], [24]]. On September 26, 2024, the U.S. Food and Drug Administration (FDA) approved xanomeline and trospium chloride capsules (trade name Cobenfy) for adults with schizophrenia [25]. Despite this success, a clear discrepancy exists between the highly screened, relatively homogeneous populations in these trials and the complex, heterogeneous patient groups found in real-world clinical practice [26,27]. This translational gap limits the generalizability and highlights the need for early post-marketing pharmacovigilance to map the complete AE spectrum and identify rare or unexpected safety signals.

The FDA Adverse Event Reporting System (FAERS) aggregates AE reports worldwide and supports real-world safety surveillance [28]. The purpose of this study is to utilize the FAERS database to mine and analyze safety signals associated with xanomeline and trospium chloride, aiming to provide early insights into the drug’s safety in routine clinical settings. Such information is clinically important for guiding clinicians and policymakers to ensure the safe and effective use of this drug.

## Materials and Methods

### Data source and collection

FAERS is a publicly accessible postmarketing pharmacovigilance database that has accumulated more than 20 million anonymized individual case safety reports (ICSRs) [29]. It compiles voluntary submissions to the FDA from pharmaceutical manufacturers, healthcare professionals, consumers, and other reporters, and therefore provides a valuable resource for postmarketing drug safety research. Data are updated quarterly and can be accessed free of charge at https://fis.fda.gov/extensions/FPD-QDE-FAERS/FPD-QDE-FAERS.html. The database comprises seven key data files: demographic and administrative information (DEMO), drug information (DRUG), adverse events (REAC), patient outcomes (OUTC), report sources (RPSR), drug therapy starts and end dates (THER), and drug indications (INDI). For this study, we extracted FAERS data from the third quarter of 2024 to the fourth quarter of 2025 to capture the most recent reports involving xanomeline and trospium chloride.

### Procedures

All reports were deduplicated to ensure record uniqueness. Data cleaning followed FDA guidance: when multiple entries shared the same CASEID, only the record with the most recent FDA_DT was retained; if both CASEID and FDA_DT were identical, the entry with the higher PRIMARYID was selected [30]. In addition, since the first quarter of 2019, each quarterly FAERS release has included a list of deleted reports, and records with CASEIDs appearing on that list were subsequently removed. To comprehensively capture all potential AE reports related to xanomeline and trospium chloride, both generic and brand names were used in the search. Spelling variations and potential typographical errors were addressed by incorporating commonly observed variants. We then restricted the dataset to reports with role_cod designated as Primary Suspect (PS) to improve analytic precision and reduce confounding. AEs were standardized according to Medical Dictionary for Regulatory Activities (MedDRA, version 28.0), mapped to Preferred Terms (PTs), and grouped by System Organ Class (SOC) to ensure consistent definitions and classifications across reports.

### Statistical analysis

Disproportionality analysis is widely used in pharmacovigilance to evaluate drug-adverse event associations by contrasting event reporting for a drug of interest with that for all other drugs in the database. For signal detection, we applied complementary frequentist and Bayesian approaches. The frequentist methods included the reporting odds ratio (ROR) and proportional reporting ratio (PRR), which are efficient for early screening because they are straightforward and relatively sensitive, although they can be less stable with sparse counts [31,32]. The Bayesian methods included the Bayesian confidence propagation neural network (BCPNN) and Multi-item gamma Poisson shrinker (MGPS). BCPNN incorporates Bayesian shrinkage to reduce random noise and yield more robust estimates [33], whereas MGPS applies empirical-Bayes shrinkage to reporting counts and can be particularly informative for rare events or small samples [34]. To strengthen robustness and limit false positive findings, particularly under sparse reporting conditions, we defined a PT as a positive AE signal for xanomeline and trospium chloride only when all four algorithms met their prespecified thresholds. Signals not documented in the approved drug label were classified as unexpected signals for further evaluation. We additionally conducted stratified analyses by sex, age group, and reporter category to explore whether the main reporting pattern was broadly preserved across subgroups and to identify potential population or reporting-source differences. These subgroup analyses were considered exploratory and were interpreted with caution. All calculations were based on 2 × 2 contingency tables presented in Supplementary Table 1, with formulas and threshold definitions provided in Supplementary Table 2. Statistical analyses were performed using R software (Version 4.5.1). To ensure methodological transparency and reproducibility, we reported this study in accordance with the READUS-PV guideline.

### Clinical priority assessment

The clinical priority assessment was designed to identify signals that may pose substantial risks to patients or public health, or that could materially shift the product benefit-risk profile and therefore merit timely risk mitigation. Emerging signals at the PT level were evaluated using a semi-quantitative framework across four domains (see Supplementary Table 3). First, clinical relevance was assessed with reference to the European Medicines Agency (EMA) lists of Important Medical Events (IMEs) and Designated Medical Events (DMEs). Second, reporting rate was characterized using the case to non case ratio and categorized in line with conventional clinical trial frequency bands as very common (≥10%), common (1–10%), and rare (≤1%). Third, signal stability reflected the consistency of disproportionality across algorithms. Fourth, the reported fatality rate was defined as the percentage of AE reports that included death. Each domain was graded into three levels and assigned 0, 1, or 2 points. AEs were then classified by total score as low (0–2), moderate (3–5), or high (6–8) clinical priority [35].

### Time-to-onset analysis

Time-to-onset (TTO) was defined as the interval between the treatment initiation date (START_DT) and the AE onset date (EVENT_DT). We excluded reports with missing dates, EVENT_DT earlier than START_DT resulting in negative TTO, or implausible onset intervals such as those exceeding the study observation window, thereby restricting analyses to records with complete and clinically plausible temporal information. TTO characteristics were summarized using the median, interquartile range, minimum, maximum, and the Weibull shape parameter. The Weibull distribution was used to model temporal changes in AE occurrence risk. Specifically, the scale parameter (α) reflects the dispersion of onset times, whereas the shape parameter (β) characterises the hazard pattern over time [36]. A decreasing hazard was inferred when β < 1 and its 95% confidence interval lay entirely below 1 (early failure-type profile). A time independent pattern was inferred when β was equal to or close to 1 and its confidence interval included 1 (random failure-type profile). An increasing hazard was inferred when β > 1 and its 95% confidence interval excluded 1 (wear-out failure-type profile). The cumulative incidence of xanomeline and trospium chloride associated AEs was visualized using Kaplan-Meier methods and compared using the log-rank test, with statistical significance defined as P < 0.05 [37].

## Results

### General characteristics

**Fig. 1:**
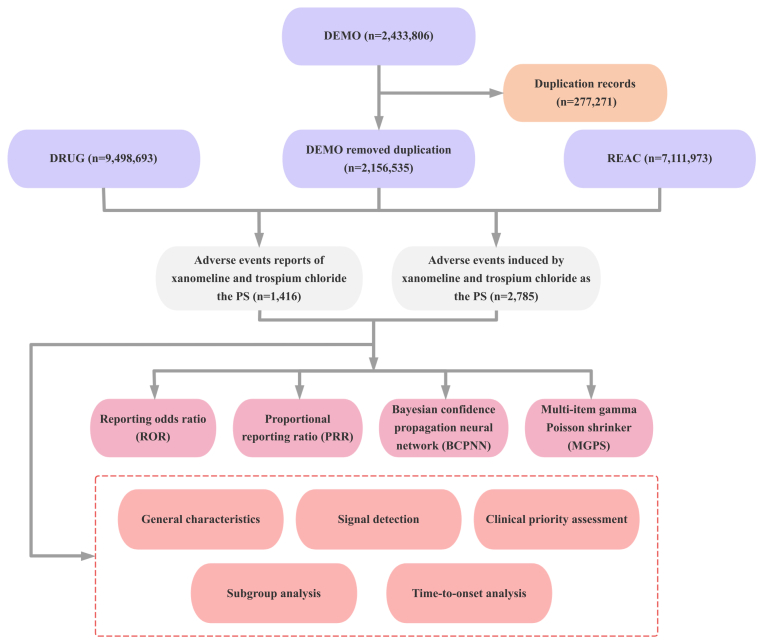
Analysis workflow of xanomeline and trospium chloride in FAERS.

From the third quarter of 2024 through the fourth quarter of 2025, a total of 2,433,806 AE reports were retrieved from the FAERS database. After deduplication, 2,156,535 reports were retained for analysis. Among these, 1416 reports listing xanomeline and trospium chloride as the primary suspected drug were identified, yielding 2785 PT records (Fig. 1). The demographic and clinical characteristics of these reports are summarized in Table 1. In the sex-stratified distribution, male patients remained the largest group (655 reports, 46.3%), followed by female patients (436 reports, 30.8%), while sex was unspecified in 325 reports (23.0%). Adults aged 18–64 years comprised the largest age band (681 reports, 48.1%), whereas older adults aged 65–85 years contributed fewer reports (57 reports, 4.0%), and patients younger than 18 years were rarely reported (7 reports, 0.5%). Age was unavailable in nearly half of reports (671 reports, 47.4%), limiting a more granular evaluation of age-related patterns. Body weight was largely unreported (1362 reports, 96.2%). Regarding reporter type, consumers (384 reports, 27.1%), other health professionals (346 reports, 24.4%), and physicians (321 reports, 22.7%) were the main sources of reports. All reports originated from the United States. For clinical outcomes, most reports did not specify an outcome (1095 reports, 77.3%). Among those with documented outcomes, other serious outcomes were most frequent (191 reports, 13.5%), followed by hospitalization (99 reports, 7.0%). Death (19 reports, 1.3%), life-threatening events (10 reports, 0.7%), and disability (2 reports, 0.1%) were uncommon. By reporting year, 156 reports (11.0%) were submitted in 2024 and 1260 (89.0%) in 2025.

**Table 1:** Demographic and clinical characteristics of adverse event reports associated with xanomeline and trospium chloride (2024 Q3 – 2025 Q4).

Characteristics	Number of events (%)
**Sex, n(%)**	
Female	436 (30.8%)
Male	655 (46.3%)
Unknown	325 (23.0%)
**Age (year), n(%)**	
<18	7 (0.5%)
18 - 64	681 (48.1%)
65 - 85	57 (4.0%)
Unknown	671 (47.4%)
**Weight, n(%)**	
<50 kg	2 (0.1%)
50–100 kg	42 (3.0%)
>100 kg	10 (0.7%)
Unknown	1362 (96.2%)
**Reporters, n(%)**	
Healthcare professional	
Physician	321 (22.7%)
Pharmacist	33 (2.3%)
Other health-professional	346 (24.4%)
Non-healthcare professional	
Consumer	384 (27.1%)
Lawyer	1 (0.1%)
Unknown	331 (23.4%)
**Reported countries, n(%)**	
United States	1416 (100%)
**Outcomes, n(%)**	
Death	19 (1.3%)
Disability	2 (0.1%)
Hospitalization	99 (7.0%)
Life-threatening	10 (0.7%)
Other serious outcomes	191 (13.5%)
Unknown	1095 (77.3%)
**Report year, n(%)**	
2024	156 (11.0%)
2025	1260 (89.0%)

### Signal detection at the SOC level

As shown in Table 2, AEs associated with xanomeline and trospium chloride spanned 23 SOCs. By report count, the five most frequently reported SOCs were gastrointestinal disorders (999 cases), psychiatric disorders (342 cases), nervous system disorders (294 cases), general disorders and administration site conditions (289 cases), and injury, poisoning and procedural complications (168 cases). Across four disproportionality methods, SOC-level signals meeting prespecified thresholds in all algorithms were observed for gastrointestinal disorders (n = 999, ROR 6.03, PRR 4.22, IC 2.08, EBGM 4.22), psychiatric disorders (n = 342, ROR 3.15, PRR 2.89, IC 1.53, EBGM 2.88), and renal and urinary disorders (n = 126, ROR 3.12, PRR 3.03, IC 1.6, EBGM 3.03). In addition, Nervous system disorders met the criterion of at least one algorithm (n = 294, ROR 1.53, PRR 1.48, IC 0.56, EBGM 1.48). Overall, these findings indicate that xanomeline and trospium chloride associated AEs cluster in specific organ systems, supporting targeted pharmacovigilance monitoring and further investigation.

**Table 2:** Distribution and signal strength of adverse events associated with xanomeline and trospium chloride at the SOC level.

System organ class (SOC)	Case Reports	ROR (95%Cl)	PRR (χ2)	EBGM(EBGM05)	IC(IC025)
Gastrointestinal disorders	999	6.03 (5.58–6.51)	4.22 (2681.95)	4.22 (3.9)	2.08 (1.97)
Psychiatric disorders	342	3.15 (2.81–3.53)	2.89 (439.64)	2.88 (2.57)	1.53 (1.35)
Nervous system disorders	294	1.53 (1.36–1.73)	1.48 (48.83)	1.48 (1.31)	0.56 (0.38)
General disorders and administration site conditions	289	0.57 (0.5–0.64)	0.61 (84.96)	0.61 (0.54)	−0.71 (−0.88)
Injury, poisoning and procedural complications	168	0.4 (0.34–0.47)	0.44 (142.93)	0.44 (0.37)	−1.2 (−1.42)
Renal and urinary disorders	126	3.12 (2.61–3.74)	3.03 (173.54)	3.03 (2.53)	1.6 (1.31)
Skin and subcutaneous tissue disorders	109	0.64 (0.53–0.78)	0.66 (21)	0.66 (0.54)	−0.61 (−0.89)
Investigations	105	0.65 (0.53–0.79)	0.66 (19.23)	0.66 (0.54)	−0.6 (−0.88)
Eye disorders	73	1.17 (0.93–1.47)	1.16 (1.73)	1.16 (0.92)	0.22 (−0.12)
Respiratory, thoracic and mediastinal disorders	41	0.3 (0.22–0.4)	0.31 (67.06)	0.31 (0.23)	−1.7 (−2.13)
Metabolism and nutrition disorders	39	0.68 (0.49–0.93)	0.68 (5.89)	0.68 (0.5)	−0.55 (−1)
Cardiac disorders	35	0.67 (0.48–0.94)	0.68 (5.58)	0.68 (0.48)	−0.57 (−1.04)
Vascular disorders	33	0.63 (0.45–0.89)	0.64 (6.96)	0.64 (0.45)	−0.65 (−1.13)
Musculoskeletal and connective tissue disorders	29	0.2 (0.14–0.29)	0.21 (92.47)	0.21 (0.14)	−2.27 (−2.76)
Surgical and medical procedures	26	0.55 (0.37–0.81)	0.55 (9.44)	0.56 (0.38)	−0.85 (−1.38)
Infections and infestations	20	0.11 (0.07–0.17)	0.12 (140.72)	0.12 (0.08)	−3.09 (−3.66)
Product issues	15	0.23 (0.14–0.38)	0.23 (38.93)	0.23 (0.14)	−2.1 (−2.76)
Reproductive system and breast disorders	12	0.67 (0.38–1.18)	0.67 (1.94)	0.67 (0.38)	−0.57 (−1.34)
Hepatobiliary disorders	7	0.25 (0.12–0.53)	0.25 (15.49)	0.25 (0.12)	−1.98 (−2.86)
Immune system disorders	7	0.2 (0.09–0.42)	0.2 (22.56)	0.2 (0.1)	−2.32 (−3.19)
Ear and labyrinth disorders	7	0.62 (0.29–1.3)	0.62 (1.66)	0.62 (0.29)	−0.69 (−1.65)
Social circumstances	6	0.4 (0.18–0.89)	0.4 (5.38)	0.4 (0.18)	−1.32 (−2.28)
Blood and lymphatic system disorders	3	0.06 (0.02–0.19)	0.06 (44.03)	0.06 (0.02)	−4.03 (−5.09)

Abbreviations: AE, adverse event; SOC, system organ class; ROR, reporting odds Ratio; PRR, proportional reporting ratio; EBGM, empirical bayesian geometric mean; IC, information component; χ2 chi-squared statistic.

### Signal detection at the PT level

**Fig. 2:**
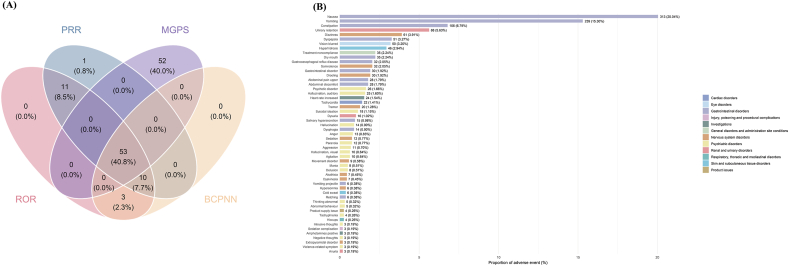
Signal detection of xanomeline and trospium chloride associated AEs at the PT level. (A) Venn diagram of overlapping PT-level signals identified by four disproportionality analysis methods. (B) Reporting counts and proportions of the 53 PT-level positive signals.

At the PT level, 53 positive signals were consistently identified across all four algorithms, with their overlap summarized in Fig. 2A. These PTs were ranked by reporting frequency in Fig. 2B and Table 3. The most frequently reported events aligned with AEs listed on the drug label, including nausea (313 cases), vomiting (239 cases), constipation (106 cases), urinary retention (88 cases), dizziness (61 cases), dyspepsia (51 cases), vision blurred (50 cases), hyperhidrosis (46 cases), dry mouth (35 cases), somnolence (32 cases), gastrooesophageal reflux disease (32 cases), and drooling (30 cases). Several unexpected signals, defined as PTs not listed on the drug label, were observed with relatively high reporting frequency, including tremor (20 cases), suicidal ideation (18 cases), dysphagia (14 cases), anger (13 cases), paranoia (12 cases), sedation (12 cases), and aggression (11 cases).

**Table 3:** Distribution and signal strength of adverse events associated with xanomeline and trospium chloride at the PT level (Ranked by number of reports).

SOC	PT	Case Reports	ROR (95%Cl)	PRR (χ2)	EBGM(EBGM05)	IC(IC025)
Gastrointestinal disorders	Nausea	313	11.19 (9.95–12.59)	10.05 (2568.93)	10.01 (8.9)	3.32 (3.11)
Gastrointestinal disorders	Vomiting	239	12.98 (11.37–14.83)	11.96 (2405.4)	11.9 (10.42)	3.57 (3.31)
Gastrointestinal disorders	Constipation	106	9.86 (8.12–11.98)	9.52 (808.97)	9.49 (7.82)	3.25 (2.85)
Renal and urinary disorders	Urinary retention	88	66.89 (53.96–82.93)	64.81 (5394.53)	63.23 (51)	5.98 (4.9)
Nervous system disorders	Dizziness	61	3.26 (2.53–4.2)	3.21 (93.22)	3.21 (2.49)	1.68 (1.26)
Gastrointestinal disorders	Dyspepsia	51	11.75 (8.91–15.52)	11.56 (490.42)	11.51 (8.72)	3.52 (2.85)
Eye disorders	Vision blurred	50	9.06 (6.85–11.99)	8.92 (350.93)	8.89 (6.72)	3.15 (2.54)
Skin and subcutaneous tissue disorders	Hyperhidrosis	46	10.56 (7.89–14.14)	10.4 (389.93)	10.36 (7.74)	3.37 (2.69)
Gastrointestinal disorders	Dry mouth	35	9.86 (7.06–13.77)	9.75 (274.09)	9.72 (6.96)	3.28 (2.48)
General disorders and administration site conditions	Treatment noncompliance	35	11.28 (8.08–15.76)	11.16 (322.53)	11.11 (7.96)	3.47 (2.63)
Nervous system disorders	Somnolence	32	4.2 (2.96–5.96)	4.16 (77.05)	4.16 (2.93)	2.06 (1.42)
Gastrointestinal disorders	Gastrooesophageal reflux disease	32	9.17 (6.46–13)	9.07 (229.31)	9.04 (6.38)	3.18 (2.36)
Nervous system disorders	Drooling	30	98.36 (68.17–141.9)	97.31 (2754.82)	93.77 (65)	6.55 (4.02)
Gastrointestinal disorders	Gastrointestinal disorder	30	5.02 (3.5–7.19)	4.97 (95.28)	4.97 (3.46)	2.31 (1.62)
Gastrointestinal disorders	Abdominal discomfort	28	3.72 (2.57–5.41)	3.7 (55.17)	3.69 (2.54)	1.88 (1.22)
Gastrointestinal disorders	Abdominal pain upper	28	3.15 (2.17–4.57)	3.13 (40.55)	3.12 (2.15)	1.64 (1)
Psychiatric disorders	Psychotic disorder	26	25.29 (17.16–37.28)	25.06 (595.08)	24.83 (16.84)	4.63 (3.16)
Psychiatric disorders	Hallucination, auditory	25	40.26 (27.08–59.87)	39.91 (934)	39.31 (26.44)	5.3 (3.42)
Investigations	Heart rate increased	24	5.94 (3.98–8.89)	5.9 (97.61)	5.89 (3.94)	2.56 (1.72)
Cardiac disorders	Tachycardia	22	6.28 (4.12–9.55)	6.23 (96.57)	6.22 (4.09)	2.64 (1.74)
Nervous system disorders	Tremor^a^	20	3.67 (2.37–5.71)	3.66 (38.6)	3.65 (2.35)	1.87 (1.06)
Psychiatric disorders	Suicidal ideation^a^	18	5.61 (3.53–8.92)	5.58 (67.58)	5.57 (3.5)	2.48 (1.5)
Renal and urinary disorders	Dysuria	16	12.01 (7.34–19.65)	11.94 (159.75)	11.89 (7.27)	3.57 (2.15)
Gastrointestinal disorders	Salivary hypersecretion	15	37.55 (22.53–62.61)	37.36 (523.17)	36.83 (22.09)	5.2 (2.78)
Gastrointestinal disorders	Dysphagia^a^	14	3.68 (2.17–6.22)	3.66 (27.09)	3.66 (2.16)	1.87 (0.89)
Psychiatric disorders	Hallucination	14	4.01 (2.37–6.79)	4 (31.45)	3.99 (2.36)	2 (0.99)
Psychiatric disorders	Anger^a^	13	14.35 (8.31–24.78)	14.29 (159.78)	14.21 (8.23)	3.83 (2.09)
Psychiatric disorders	Paranoia^a^	12	21.78 (12.33–38.5)	21.69 (234.93)	21.52 (12.18)	4.43 (2.25)
Nervous system disorders	Sedation^a^	12	7.87 (4.46–13.89)	7.84 (71.48)	7.82 (4.43)	2.97 (1.56)
Psychiatric disorders	Aggression^a^	11	7.35 (4.06–13.3)	7.32 (59.92)	7.31 (4.04)	2.87 (1.42)
Psychiatric disorders	Agitation	10	4.62 (2.48–8.6)	4.61 (28.2)	4.6 (2.47)	2.2 (0.92)
Psychiatric disorders	Hallucination, visual	10	10.04 (5.39–18.69)	10 (80.74)	9.97 (5.35)	3.32 (1.58)
Nervous system disorders	Movement disorder	9	7.65 (3.97–14.73)	7.62 (51.67)	7.61 (3.95)	2.93 (1.28)
Psychiatric disorders	Delusion^a^	8	11.48 (5.72–23.01)	11.45 (75.95)	11.4 (5.69)	3.51 (1.44)
Psychiatric disorders	Mania^a^	8	16.02 (7.99–32.14)	15.98 (111.66)	15.89 (7.92)	3.99 (1.61)
Nervous system disorders	Dyskinesia	7	5.07 (2.41–10.65)	5.06 (22.77)	5.05 (2.4)	2.34 (0.72)
Nervous system disorders	Akathisia^a^	7	14.29 (6.79–30.07)	14.26 (85.85)	14.19 (6.74)	3.83 (1.4)
Gastrointestinal disorders	Retching	6	6.86 (3.07–15.29)	6.84 (29.87)	6.83 (3.06)	2.77 (0.8)
Skin and subcutaneous tissue disorders	Cold sweat^a^	6	9.51 (4.26–21.23)	9.5 (45.45)	9.46 (4.24)	3.24 (1)
Nervous system disorders	Hypersomnia	6	5.22 (2.34–11.63)	5.21 (20.36)	5.2 (2.33)	2.38 (0.61)
Gastrointestinal disorders	Vomiting projectile	6	37.34 (16.67–83.68)	37.27 (208.72)	36.74 (16.4)	5.2 (1.49)
Psychiatric disorders	Abnormal behavior^a^	5	6.15 (2.55–14.79)	6.14 (21.45)	6.12 (2.54)	2.61 (0.54)
Psychiatric disorders	Thinking abnormal^a^	5	10.18 (4.23–24.52)	10.16 (41.15)	10.13 (4.2)	3.34 (0.82)
Respiratory, thoracic and mediastinal disorders	Hiccups^a^	4	11.89 (4.45–31.77)	11.87 (39.65)	11.82 (4.42)	3.56 (0.61)
Psychiatric disorders	Tachyphrenia^a^	4	36.65 (13.65–98.4)	36.6 (136.55)	36.09 (13.44)	5.17 (0.87)
Product issues	Product supply issue	4	7.09 (2.66–18.93)	7.08 (20.84)	7.06 (2.65)	2.82 (0.38)
Renal and urinary disorders	Anuria	3	11.44 (3.68–35.59)	11.43 (28.43)	11.38 (3.66)	3.51 (0.21)
Psychiatric disorders	Violence-related symptom^a^	3	114.42 (35.97–364)	114.3 (322.49)	109.44 (34.4)	6.77 (0.48)
Nervous system disorders	Extrapyramidal disorder	3	7.1 (2.28–22.05)	7.09 (15.65)	7.07 (2.28)	2.82 (0.04)
Psychiatric disorders	Negative thoughts^a^	3	26.25 (8.41–81.92)	26.23 (72.06)	25.97 (8.32)	4.7 (0.39)
Investigations	Amphetamines positive^a^	3	283.93 (86.09–936.49)	283.63 (760.41)	255.37 (77.42)	8 (0.45)
Injury, poisoning and procedural complications	Sedation complication^a^	3	19.21 (6.17–59.86)	19.19 (51.35)	19.06 (6.12)	4.25 (0.34)
Psychiatric disorders	Intrusive thoughts^a^	3	29.26 (9.37–91.37)	29.23 (80.87)	28.91 (9.26)	4.85 (0.4)

Abbreviations: AE, adverse event; SOC, system organ class; PT, preferred term; ROR, reporting odds Ratio; PRR, proportional reporting ratio; EBGM, empirical bayesian geometric mean; IC, information component; χ2 chi-squared statistic.

a Indicates unexpected signals, defined as signals not listed in the drug label.

These PT signals were ranked by EBGM in the Supplementary Table 4. Prominent label-consistent findings included drooling (n = 30, ROR 98.36, PRR 97.31, IC 6.55, EBGM 93.77), urinary retention (n = 88, ROR 66.89, PRR 64.81, IC 5.98, EBGM 63.23), and hallucination, auditory (n = 25, ROR 40.26, PRR 39.91, IC 5.30, EBGM 39.31). Rare but high signal-strength unexpected signals were also detected, including amphetamines positive (n = 3, ROR 283.93, PRR 283.63, IC 8, EBGM 255.37), violence-related symptom (n = 3, ROR 114.42, PRR 114.3, IC 6.77, EBGM 109.44), tachyphrenia (n = 4, ROR 36.65, PRR 36.6, IC 5.17, EBGM 36.09), intrusive thoughts (n = 3, ROR 29.26, PRR 29.23, IC 4.85, EBGM 28.91), and negative thoughts (n = 3, ROR 26.25, PRR 26.23, IC 4.7, EBGM 25.97), among others. Reporting frequencies describe the distribution of coded AE terms, whereas disproportionality metrics quantify relative reporting imbalance against the background and should not be interpreted as incidence or evidence of causality.

### Clinical priority assessment

To more precisely appraise the clinical relevance of the identified positive signals, we used a semi-quantitative scoring framework to prioritize clinically important PT signals, drawing on the EMA designated lists of DMEs and IMEs. Among the 53 positive signals, 21 (39.6%) were assigned a moderate clinical priority, whereas 32 (60.4%) were classified as low priority, and none met the criteria for high clinical priority. Six signals were categorized as Important Medical Events, and all were assigned a moderate clinical priority, including urinary retention (n = 88, score: 4), hallucination, auditory (n = 25, score: 3), suicidal ideation (n = 18, score: 3), hallucination (n = 14, score: 3), hallucination, visual (n = 10, score: 3), and anuria (n = 3, score: 3). The complete list of all positive signals, together with individual and total scores, is provided in the Supplementary Table 5.

### Subgroup-stratified safety profiles

Subgroup-stratified analyses applied the same four disproportionality algorithms as the primary analysis. For brevity, ROR values are reported in the text, and full results for all algorithms are provided in Supplementary Tables 6–8.

Sex stratification showed substantial overlap in the positive signal spectrum at the PT level, with 42 PTs in males and 27 in females, including 22 shared PTs, while the remaining PTs showed sex-specific predominance. The shared profile was driven mainly by gastrointestinal intolerance and anticholinergic type events, including nausea, vomiting, constipation, urinary retention, dyspepsia, dry mouth, hyperhidrosis, and vision blurred. Within the shared set, females showed stronger disproportionality for drooling (ROR = 217.95), salivary hypersecretion (ROR = 72.91), and hallucination related PTs including hallucination, auditory (ROR = 53.66) and hallucination, visual (ROR = 14.18), whereas dry mouth (ROR = 19.77) and dyspepsia (ROR = 15.84) were more disproportionate in males. Sex-specific PTs were limited in females and included somnolence, tremor, suicidal ideation, akathisia, and vomiting projectile, whereas males showed a broader set of unique PTs spanning neuropsychiatric and reporting or product related terms, with selected examples including aggression, agitation, delusion, abdominal pain upper, insomnia, and amphetamines positive.

Age stratification identified 40 PTs in adults aged 18–64 years and 10 in those aged 65–85 years, with nine shared PTs. Across both age bands, report counts were led by nausea and vomiting, while the highest RORs in adults aged 18–64 years were observed for drooling (ROR = 150.18) and urinary retention (ROR = 101.29). In older adults, urinary retention showed the highest disproportionality (ROR = 63.71) and dry mouth (ROR = 23.88) and dysuria (ROR = 32.67) were relatively more prominent, with feeling abnormal detected only in this stratum (ROR = 9.60).

Reporter type stratification identified 38 PTs in healthcare professional reports and 34 in non healthcare reports, with 21 shared PTs. The overall spectrum was broadly comparable, but the emphasis differed across reporter groups. Non-healthcare professional reports more often highlighted subjective symptoms and treatment outcome coding, including sedation (ROR = 23.54), hyperhidrosis (ROR = 16.59), dyspepsia (ROR = 12.54), therapy non-responder (ROR = 9.88), and therapeutic response decreased (ROR = 9.36). In contrast, healthcare professional reports more frequently captured clinically salient PTs, including blood pressure increased (ROR = 4.15), heart rate increased (ROR = 13.47), angioedema (ROR = 5.11), intestinal obstruction (ROR = 6.51), and amphetamines positive (ROR = 954.42), with suicidal ideation also detected (ROR = 8.04).

### Time-to-onset analysis

**Fig. 3:**
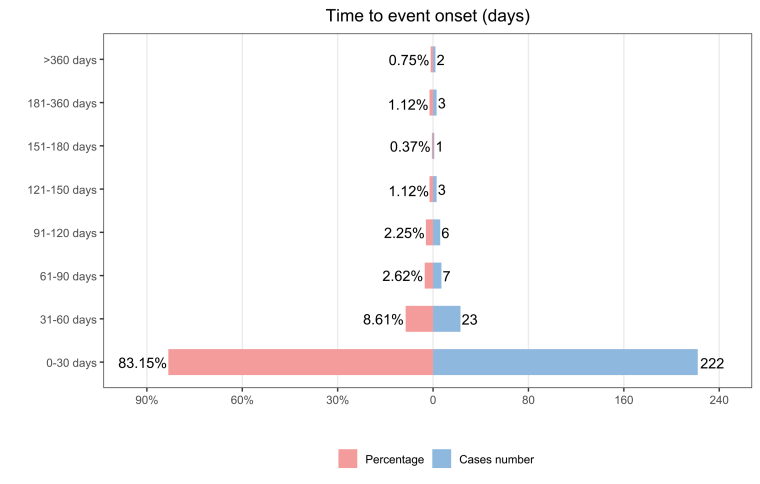
Time-to-onset distribution of adverse events associated with xanomeline and trospium chloride. Most events occurred within 0–30 days after treatment initiation, with case counts and percentages shown for each onset interval.

**Fig. 4:**
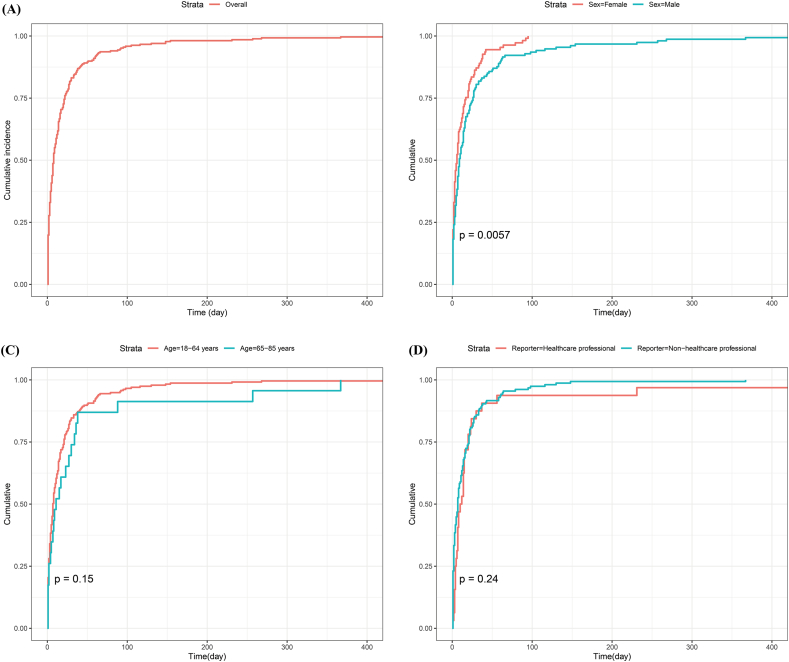
Cumulative incidence curves for time to onset of adverse events associated with xanomeline and trospium chloride. (A) Overall time-to-onset distribution. (B) Sex-stratified comparison (female vs male). (C) Age-stratified comparison (18–64 vs 65–85 years). (D) Reporter-type comparison (healthcare professional vs non-healthcare professional).

After excluding reports with implausible dates, missing information, or an indeterminate onset date, 267 cases were retained for the TTO analysis. Most AEs occurred within the first month after initiation of xanomeline and trospium chloride (n = 222, 83.15%), and the number of events declined steadily from the second month onward (Fig. 3; Fig. 4A). The overall median TTO was 8 days (IQR 2–20, range 1–148). Weibull distribution analysis showed that the upper bound of the 95% confidence interval for the shape parameter β remained below 1, supporting an early failure pattern with a declining hazard over time (Table 4).

**Table 4:** Time-to-onset analysis of xanomeline and trospium chloride using the Weibull distribution test.

Categories	Cases, n	TTO (days)		Weibull distribution				Failure Type
				Scale parameter		Shape parameter		
		Median (IQR)	Min - Max	α	95% CI	β	95% CI	
**Overall**	267	8 (2–22)	1–437	16.18	13.11–19.24	0.67	0.61–0.73	Early failure
**Sex**								
Female	109	6 (2–17)	1–95	11.48	8.51–14.44	0.77	0.66–0.88	Early failure
Male	154	9 (3–26)	1–437	19.76	14.72–24.81	0.66	0.58–0.73	Early failure
**Age**								
18–64 years	235	7 (2–21)	1–437	15.05	12.09–18.01	0.69	0.63–0.75	Early failure
65–85 years	23	11 (3–32)	1–367	25.68	7.02–44.33	0.60	0.42–0.77	Early failure
**Reporter**								
Healthcare professional	32	11 (4.5–20)	1–437	21.85	9.84–33.87	0.67	0.52–0.83	Early failure
Non-healthcare professional	156	7 (2–21)	1–367	13.40	10.20–16.59	0.70	0.62–0.78	Early failure

Abbreviations: TTO,Time-to-onset; α, Scale parameter; β, Shape parameter.IQR, interquartile range; CI, confidence interval; n, number of cases with available time-to-onset.

Subgroup comparisons indicated that the TTO distribution differed mainly by sex, whereas age and reporter type showed broadly similar patterns. Females experienced earlier AE onset than males (median TTO [IQR] 6 days [[2], [3], [4], [5], [6], [7], [8], [9], [10], [11], [12], [13], [14], [15], [16], [17]] vs 9 days [[3], [4], [5], [6], [7], [8], [9], [10], [11], [12], [13], [14], [15], [16], [17], [18], [19], [20], [21], [22], [23], [24], [25], [26]], log-rank test p = 0.0057, Fig. 4B). Age strata showed no clear separation (18–64 vs 65–85 years, median TTO [IQR] 7 days [[2], [3], [4], [5], [6], [7], [8], [9], [10], [11], [12], [13], [14], [15], [16], [17], [18], [19], [20], [21]] vs 11 days [[3], [4], [5], [6], [7], [8], [9], [10], [11], [12], [13], [14], [15], [16], [17], [18], [19], [20], [21], [22], [23], [24], [25], [26], [27], [28], [29], [30], [31], [32]], log-rank test p = 0.15, Fig. 4C). Reporter type curves were also comparable (healthcare professional vs non healthcare professional, median TTO [IQR] 11 days [4.5–20] vs 7 days [[2], [3], [4], [5], [6], [7], [8], [9], [10], [11], [12], [13], [14], [15], [16], [17], [18], [19], [20], [21]], log-rank test p = 0.24, Fig. 4D). Across strata, Weibull shape parameters remained below 1, consistent with early clustering of events and a declining hazard over time.

## Discussion

As the first FDA-approved oral cholinergic therapy for adult schizophrenia in decades, xanomeline and trospium chloride marks a mechanistic shift. Using real-world FAERS data, we provide complementary evidence on its clinical safety.

### Demographic profile and generalizability of findings

We identified 1416 AE reports listing xanomeline and trospium chloride as the primary suspected drug, providing an early postmarketing perspective following its late 2024 introduction. Reporting increased markedly in 2025 (89.0%), consistent with expanding clinical uptake during the initial launch period. Sources were diverse, with consumers contributing 27.1% of reports, while physicians (22.7%) and other health professionals (24.4%) together accounted for nearly half, which supports active pharmacovigilance and richer clinical detail. Among cases with available sex information, males represented 46.3% and females 30.8%, a distribution that more closely reflects the epidemiology of schizophrenia, characterized by slightly higher incidence and earlier onset in men, than any confirmed difference in AE susceptibility [38,39]. Adults aged 18–64 years comprised 48.1% of reports, aligning with the primary treatment population for schizophrenia, whereas older adults (4.0%) and pediatric patients (0.5%) were underrepresented. All reports originated from the United States (100%), consistent with the launch market but limiting extrapolation to other regions. Serious outcomes, including hospitalization (7.0%) and death (1.3%), were reported but remained infrequent within this early dataset, and most cases lacked clearly documented outcomes (77.3%), warranting cautious interpretation.

### Mechanism-anchored interpretation of principal signals

#### Gastrointestinal disorders

Gastrointestinal AEs are a key tolerability concern in cholinergic treatment for schizophrenia given their potential to compromise adherence [40]. In our analysis, gastrointestinal disorders were the strongest and most frequently reported SOC-level signal (n = 999, ROR 6.03, PRR 4.22, IC 2.08, EBGM 4.22). The observed pattern included both cholinergic-predominant events, such as nausea and vomiting, and anticholinergic-predominant events, such as constipation and dyspepsia, which is concordant with the approved prescribing information and phase 3 trial data, in which these gastrointestinal reactions were among the most frequently reported AEs [24]. This pattern is pharmacologically plausible because xanomeline-mediated M1 and M4 receptor agonism is intended to be counterbalanced by the peripherally acting muscarinic antagonist trospium chloride. The prescribing information further indicates that this balance is sensitive to food intake, recommending administration at least 1 h before meals or at least 2 h after meals [41]. Under fed conditions, trospium exposure is markedly reduced, with Cmax decreasing by 70%–75% and AUC by 85%–90%, while a high-fat meal increases xanomeline exposure by about 30%. Taken together, these findings support clear counseling on fasting administration, with gastrointestinal intolerance managed through symptomatic treatment and cautious dose adjustment rather than premature discontinuation. Given the predominance of gastrointestinal AEs early after treatment initiation, slower titration, delayed dose escalation, or temporary dose reduction may help reduce gastrointestinal AEs in patients who develop early nausea, vomiting, or constipation.

#### Renal and urinary disorders

Renal and urinary AEs represent another clinically relevant signal because they can precipitate acute urinary retention and infection and may interrupt treatment. Renal and urinary disorders showed a robust SOC-level signal (n = 126, ROR 3.12, PRR 3.03, IC 1.6, EBGM 3.03). At the PT-level, urinary retention was one of the most prominent signals (n = 88, ROR 66.89, PRR 64.81, IC 5.98, EBGM 63.23). This pattern is biologically plausible because normal detrusor contraction depends predominantly on muscarinic M3 receptor signaling [42], and peripheral antimuscarinic activity may impair bladder emptying, particularly in patients with bladder outlet obstruction or incomplete voiding [43]. Trospium chloride pharmacokinetics further support this interpretation, as the drug is substantially excreted by the kidney and systemic exposure is increased in renal impairment, which may heighten the risk of anticholinergic AEs, including urinary retention and urinary tract infection [44]. Clinically, these findings suggest that urinary symptoms should be actively assessed before and shortly after treatment initiation, particularly in older adults and patients with pre-existing urinary retention or impaired bladder emptying. Early attention to hesitancy, reduced urinary stream, incomplete bladder emptying, or painful urination may help clinicians identify worsening urinary symptoms before they become more severe or interfere with treatment continuation.

#### Other signals of interest

Beyond the dominant gastrointestinal and renal-urinary findings, several additional PT signals further refined the early postmarketing safety profile of xanomeline and trospium chloride. Several prominent signals identified in this analysis were broadly consistent with AEs described in the prescribing information, particularly dizziness, blurred vision, somnolence, drooling, and heart rate-related events, including increased heart rate and tachycardia, thereby supporting the clinical plausibility of these findings. These events may reflect the combined influence of central muscarinic engagement and peripheral autonomic effects, making early assessment of alertness, balance, visual symptoms, and vital signs particularly relevant during treatment initiation and titration. Hyperhidrosis was also notable and may represent a related autonomic manifestation, given the cholinergic control of eccrine sweat secretion [45]. Movement-related terms, including tremor, akathisia, dyskinesia, and movement disorder, are mechanistically interpretable in light of the established role of M1 and M4 receptor signaling, together with striatal cholinergic interneurons, in shaping dopaminergic transmission and basal ganglia plasticity [46]. These observations should nevertheless be interpreted cautiously, as abnormal motor phenomena in schizophrenia may also be influenced by baseline disease features, prior antipsychotic exposure, and concomitant medications [47]. Several additional signals not listed in the prescribing information were also observed, including sedation, cold sweat, hiccups, amphetamines positive, and sedation complication, and these are better regarded as exploratory than confirmatory. Among these, sedation-related terms are directionally compatible with the recognized central nervous system effects of this combination, although sedation complication may more plausibly reflect downstream clinical context than a primary pharmacologic effect [48]. Cold sweat may likewise be consistent with autonomic cholinergic involvement in sweat regulation [49], whereas hiccups have a biologically conceivable basis given the participation of vagal, phrenic, and sympathetic pathways in the hiccup reflex arc, although attribution to a direct drug effect remains uncertain [50]. By contrast, terms such as amphetamines positive are more likely to reflect co-exposure or reporting context than a reproducible drug-specific toxicity. Overall, these observations suggest that central nervous system, autonomic, and motor symptoms should be actively monitored during treatment initiation and dose escalation. Particular attention to somnolence, dizziness, blurred vision, sweating, abnormal movements, and heart rate changes may help clinicians identify emerging tolerability problems early and intervene before these symptoms compromise adherence or treatment continuation.

### Shifting the safety paradigm: a comparative analysis against D_2_ antagonists

Xanomeline and trospium chloride, the first approved M1 and M4 muscarinic agonist, offers value through efficacy and through a risk–benefit profile that departs from dopamine D2 antagonists. Our signal detection revealed a pattern dominated by gastrointestinal intolerance and peripheral anticholinergic events, supporting a meaningful shift in the principal domains of safety concern. We found no strong signals for weight gain or metabolic disturbance, consistent with the metabolically neutral trend observed in clinical trials and in contrast to the substantial weight-gain liability reported with high-risk second-generation antipsychotics, particularly olanzapine and clozapine, with clinically relevant weight gain reported in up to 72% of antipsychotic-treated patients overall [51,52]. Because this regimen does not directly block dopaminergic pathways, it also appears to largely avoid hyperprolactinemia [53]. Although several movement-related PTs were detected, these signals were less prominent than the dominant gastrointestinal and urinary events and were not accompanied by a distinct system-level extrapyramidal profile. This pattern is broadly aligned with the prescribing information, in which non-akathisia extrapyramidal symptoms are recognized but do not constitute a major safety emphasis [41]. The cardiovascular profile also appears to differ, with a mean increase in heart rate of approximately 9.8 beats per minute, whereas QTc prolongation remains a more prominent concern with certain atypical antipsychotics, particularly higher-risk agents such as ziprasidone and iloperidone [54]. AEs clustered primarily in the gastrointestinal domain, with nausea, vomiting, and constipation most often reported. In the broader clinical context, this pattern differs from the severe gastrointestinal hypomotility associated with clozapine. Overall, these findings suggest that post-initiation monitoring for xanomeline and trospium chloride should place particular emphasis on gastrointestinal tolerability, urinary retention risk, central anticholinergic effects, and heart rate surveillance, while routine metabolic and neurologic monitoring remains clinically appropriate.

### Distinguishing drug effect from disease manifestation: psychiatric AEs and bias

Psychiatric disorders constituted a prominent signal domain (n = 342, ROR 3.15, PRR 2.89, IC 1.53, EBGM 2.88), but interpretation requires caution because this category likely captures both illness-related phenomena and a subset of pharmacologically plausible central anticholinergic effects. Leading PTs such as psychotic disorder and paranoia overlap substantially with the core positive psychopathology and symptom fluctuation of schizophrenia, whereas suicidal ideation may be more reflective of illness severity or affective burden than de novo drug toxicity [55,56]. By contrast, hallucination-related terms may be more biologically interpretable in some cases, as the prescribing information specifically notes that trospium chloride is associated with anticholinergic central nervous system effects, including confusion, hallucinations, and somnolence, particularly after treatment initiation or dose escalation [41]. This distinction is further complicated by indication bias and the symptom-driven nature of spontaneous reporting, both of which may enrich psychiatric terms in patients with greater baseline severity. Overall, psychiatric PTs in this setting are best interpreted as a mixed signal domain requiring case-level clinical contextualization.

### Robustness of the safety profile and subgroup heterogeneity

Subgroup analyses largely preserved the primary signal architecture, as the shared spectrum across sex, age, and reporter strata continued to be dominated by gastrointestinal intolerance and anticholinergic-type events, supporting the overall robustness of the principal safety profile. At the same time, several female-specific AEs were identified, including somnolence, tremor, akathisia, and projectile vomiting, whereas male-specific signals were also observed, such as insomnia, abdominal pain upper, retching, cold sweating, and aggression. However, the sex-stratified findings should be interpreted as signals for future investigation rather than evidence of established sex-specific risk. These differences may be broadly consistent with established sex-related variation in drug disposition and tolerability, as women often differ from men in body weight, body fat composition, renal clearance, and cytochrome P450-dependent metabolism, all of which can influence drug absorption, distribution, metabolism, and elimination and may thereby alter systemic exposure to psychotropic agents [[57], [58], [59]]. Prior pharmacokinetic evidence further suggests that, at equivalent doses, women frequently exhibit higher plasma drug concentrations or longer elimination times, a pattern that may contribute to sex-specific differences in adverse-event burden and clinical tolerability [60]. In the age-stratified analysis, nausea and vomiting were common across all age groups, whereas urinary retention, dysuria, and dry mouth were relatively more prominent in older adults, a pattern consistent with the greater susceptibility of later-life populations to peripheral anticholinergic effects [61,62]. Differences across reporter types also appeared to reflect reporting tendencies rather than intrinsic biological risk: non-healthcare reports more often emphasized subjective symptoms and treatment-response terms, whereas healthcare professional reports more frequently documented clinically important events such as tachycardia, angioedema, and intestinal obstruction. Taken together, these subgroup findings reinforce the consistency of the main signals while also indicating that patient characteristics and reporting source may influence the recognition and recording priority of specific AEs.

### Temporal dynamics and monitoring

Because early tolerability often influences treatment continuation, prompt recognition and management of AEs are essential. In this study, the risk profile of xanomeline and trospium chloride was distinctly front-loaded: most AEs occurred within the first month, the median TTO was 8 days, and Weibull analysis consistently indicated an early failure pattern. These findings suggest that risk is highest soon after initiation and declines thereafter, rather than accumulating with continued exposure. This temporal pattern is consistent with the EMERGENT program, in which cholinergic and anticholinergic AEs typically emerged within the first 1–2 weeks and were often transient [63]. Women showed earlier onset than men, whereas differences by age and reporter type were less pronounced. These temporal findings indicate that the first month after treatment initiation is a key period for tolerability management, particularly in women and in patients vulnerable to gastrointestinal intolerance. Early follow-up after initiation or dose escalation may allow clinicians to reinforce fasting administration, review gastrointestinal and urinary symptoms, assess dizziness, somnolence, visual symptoms, and pulse changes, and adjust the titration pace when early tolerability problems emerge.

### Limitations

When interpreting the present study, several limitations inherent to the FAERS database warrant particular attention. First, because FAERS is a spontaneous reporting system, its data cannot support causal inference, and disproportionality analyses indicate statistical associations rather than causality [64]. Accordingly, any detected signals should be regarded as hypotheses for further validation rather than definitive evidence of risk. Second, the dataset is subject to underreporting, duplicate records, variable data quality, and the absence of exposure denominators, which precludes estimation of true incidence rates or direct risk comparisons. Third, although we strengthened the robustness of the analysis by applying four disproportionality methods and restricting the dataset to reports in which xanomeline and trospium chloride were designated as the primary suspected drugs, residual confounding related to disease severity, comorbidities, and concomitant therapies cannot be fully excluded. In addition, all reports were derived from the United States, which substantially limits the external generalizability of the findings. Taken together, these results identify potential safety signals associated with xanomeline and trospium chloride in real-world use. However, this safety profile should be updated through continued FAERS monitoring and, where feasible, validated using active surveillance systems, electronic health records, or prospective real-world cohort studies, thereby strengthening the evidence base for informed clinical practice.

## Patient Consent

Not applicable.

## Ethical approval

Ethical approval was not required for this study.

## Data availability

The datasets supporting this study’s findings were sourced from the FAERS database, accessible via: https://fis.fda.gov/extensions/FPD-QDE-FAERS/FPD-QDE-FAERS.html.

## Author contributions

Zhuocheng Bao and Yakun Liu: Designed this study and Original protocol, Methodology. Zhuocheng Bao: Data curation, Formal analysis, and Writing-original draft. Yakun Liu and Yuxiang Liu: Supervision. Yuxiang Liu: Resources, Writing-review & editing, and Project administration.

## Funding

None declared.

## Declarations of competing interest

None.
