# Supplementary material for: Safety profile and potential clinical risks of xanomeline and trospium chloride: A real-world pharmacovigilance study using FAERS
Source: Neurotherapeutics. 2026 May 27;23(4):e00930. doi: 10.1016/j.neurot.2026.e00930 (PMC13234482; doi:10.1016/j.neurot.2026.e00930)
Supplement: Multimedia component 1 [file mmc1.docx]

**Supplementary Table 1.** Two-by-two contingency table for disproportionality analyses.

|  | Target adverse event | Non-target adverse events | Total |
| --- | --- | --- | --- |
| Target drug | a | b | a+b |
| Non-target drugs | c | d | c+d |
| Total | a+c | b+d | a+b+c+d |

Abbreviations: a, number of target adverse event for the target drug; b, number of non-target adverse events for the target drug; c, number of target adverse event for non-target drugs; d, number of non-target adverse events for non-target drugs.
