# Supplementary material for: Safety profile and potential clinical risks of xanomeline and trospium chloride: A real-world pharmacovigilance study using FAERS
Source: Neurotherapeutics. 2026 May 27;23(4):e00930. doi: 10.1016/j.neurot.2026.e00930 (PMC13234482; doi:10.1016/j.neurot.2026.e00930)
Supplement: Multimedia component 2 [file mmc2.docx]

**Supplementary Table 2.** Four major algorithms used for signal detection.

| **Algorithms** | **Equation** | **Criteria** |
| --- | --- | --- |
| ROR | ROR=ad/bc | lower limit of 95% CI>1, N≥3 |
|  | 95%CI=e^ln(ROR)±1.96(1/a+1/b+1/c+1/d)^0.5^ |  |
| PRR | PRR=a(c+d)/c(a+b) | PRR≥2, χ^2^≥4, N≥3 |
|  | χ^2^=[(ad-bc)^2](a+b+c+d)/[(a+b)(c+d)(a+c)(b+d)] |  |
| MGPS | EBGM=a(a+b+c+d)/(a+c)(a+b) | EBGM05>2 |
|  | 95%CI=e^ln(EBGM)±1.96(1/a+1/b+1/c+1/d)^0.5^ |  |
| BCPNN | IC=log_2_a(a+b+c+d)/(a+c)(a+b) | IC025>0 |
|  | 95%CI= E(IC) ± 2V(IC)^0.5 |  |

Abbreviations: a, number of target adverse event for the target drug; b, number of non-target adverse events for the target drug; c, number of target adverse event for non-target drugs; d, number of non-target adverse events for non-target drugs. ROR, reporting odds ratio; PRR, proportional reporting ratio; BCPNN, bayesian confidence propagation neural network; MGPS, multi-item gamma Poisson shrinker; EBGM, empirical Bayesian geometric mean; 95%CI, 95% confidence interval; N, the number of reports; χ2, chi-squared; IC, information component; IC025, the lower limit of 95% CI of the IC; E(IC), the IC expectations; V(IC), the variance of IC; EBGM, empirical Bayesian geometric mean; EBGM05, the lower limit of 95% CI of EBGM.
