# Supplementary material for: Safety profile and potential clinical risks of xanomeline and trospium chloride: A real-world pharmacovigilance study using FAERS
Source: Neurotherapeutics. 2026 May 27;23(4):e00930. doi: 10.1016/j.neurot.2026.e00930 (PMC13234482; doi:10.1016/j.neurot.2026.e00930)
Supplement: Multimedia component 3 [file mmc3.docx]

**Supplementary Table 3.** Criteria and corresponding semi-quantitative scoring system for prioritizing adverse drug events identified through disproportionality analysis.

| **Criterium** | **2 points** | **1 point** | **0 point** |
| --- | --- | --- | --- |
| Reporting rate (cases/noncases) | >10% | 1% - 10% | 0 - 1% |
| Signal stability (consistency across disproportionality analyses) | 3 of 3 | 2 of 3 | 1 of 3 |
| Reported case fatality rate (proportion of reports with death as outcome) | >50% | 25-50% | <25% |
| Clinical relevance (serious likely drug-attributable AEs) | DME | IME | None |

Abbreviations: AEs, adverse events; DME, designated medical event; IME, important medical event.
